# Pharmacokinetics and Immunogenicity of Broadly Neutralizing HIV Monoclonal Antibodies in Macaques

**DOI:** 10.1371/journal.pone.0120451

**Published:** 2015-03-25

**Authors:** Yvonne Rosenberg, Markus Sack, David Montefiori, Celia Labranche, Mark Lewis, Lori Urban, Lingjun Mao, Rainer Fischer, Xiaoming Jiang

**Affiliations:** 1 PlantVax Corporation, Rockville, Maryland, United States of America; 2 Institute of Molecular Biotechnology, RTWH Aachen University, Aachen, Germany; 3 Department of Surgery, Duke University Medical Center, Durham, North Carolina, United States of America; 4 Bioqual Inc., Rockville, Maryland, United States of America; Shanghai Medical College, Fudan University, CHINA

## Abstract

The identification of highly potent broadly neutralizing antibodies (bnAbs) against HIV-1, and success in preventing SHIV infection following their passive administration, have increased the likelihood that immunotherapeutic strategies can be adopted to prevent and treat HIV-1 infection. However, while broad and potent neutralizing activity is an essential prerequisite, in vivo properties such as good circulatory stability and non-immunogenicity are equally critical for developing a human treatment. In the present study, glycoforms of the bnAbs 10-1074, NIH45-46^G54W^, 10E8, PGT121, PGT128, PGT145, PGT135, PG9, PG16, VRC01 and b12 were produced by *Agrobacterium*-mediated transient transfection of *Nicotiana benthamiana* and assessed following administration in rhesus macaques. The results indicate that (i) N-glycans within the VL domain impair plasma stability of plant-derived bnAbs and (ii) while PGT121 and b12 exhibit no immunogenicity in rhesus macaques after multiple injections, VRC01, 10-1074 and NIH45-46^G54W^ elicit high titer anti-idiotypic antibodies following a second injection. These anti-idiotypic antibodies specifically bind the administered bnAb or a close family member, and inhibit the bnAb in neutralization assays. These findings suggest that specific mutations in certain bnAbs contribute to their immunogenicity and call attention to the prospect that these mutated bnAbs will be immunogenic in humans, potentially compromising their value for prophylaxis and therapy of HIV-1.

## Introduction

Developments in large scale screening for HIV+ individuals producing broadly neutralizing HIV antibodies, together with efficient single cell antibody cloning techniques, have led to the identification of increasingly potent HIV bnAbs [1–3]. Since protection against challenge with chimeric simian-HIV (SHIV) isolates through the use of first-generation bnAb cocktails has previously been achieved in macaques [4–7], the availability of bnAbs with superior neutralizing properties greatly increases the prospect that therapeutic strategies involving passive immunotherapy will find application in preventing infection in humans in the case of mother-to-child transmission, sexual transmission and in controlling both acute and chronic infections [8–11].

The HIV envelope epitopes of these potent and broadly neutralizing antibodies generally fall into several categories: those predominantly targeting either the CD4 binding site (CD4bs), epitopes partly comprising carbohydrates on the gp120 [12–16], the membrane proximal external region (MPER) and an epitope spanning both gp120 and gp41 [17,18]. Within the family of glycan epitopes, subgroups are becoming evident, although almost all mAbs are directed towards oligomannose glycans e.g. (i) high mannose epitopes on the V1/V2 variable loop (PG9/PG16) and (ii) the N332A sensitive complex glycan on the V3 loop (2G12, PGTs, 10–1074). In the latter group, minor differences may lead to marked changes in potency. Thus, while PGT128 interacts with two oligomannose glycans N301 and N332 as well as with the base of the V3 loop, the more potent PGT121 mAb appears more dependent on N332 than N301, and uniquely recognizes a complex glycan epitope terminating in galactose or α2–6-linked sialic acid [19, 20].

While high in vitro neutralization potency is a prerequisite for an antibody’s ability to passively protect against or control HIV in vivo, its therapeutic potential will also depend on its plasma stability and immunogenicity, as well as ease and cost of production. Antibodies against therapeutics are frequently observed, and have important clinical implications such as accelerated drug clearance and neutralization. In the context of passive mAb treatment, the development of anti-drug antibodies, e.g. against adalimumab, has been associated with lower mAb concentration and loss of efficacy of the drug [20]. This potential challenge, in addition to the rapid emergence of viral escape mutants in infected recipients, may necessitate constant development of new potent antibody-based therapies on an on-going basis to counteract both viral resistance and anti-drug antibodies. In this context, plant-based transient expression systems offer unique advantages in their speed, versatility, pathogen-free nature and low-tech requirements, in particular in the early developmental stages from “cloning to preclinical protection studies” [21–23].

Recently, we have shown that plant-derived HIV-1 mAbs 2F5, 4E10, b12, and VRC01 produced at high levels in the transient *N*.*benthamiana/p19* (*Nb*/p19) system, exhibit potency and functional properties similar to their mammalian cell counterparts [24]. In the present study, the transient plant *Nb*/p19 system has been used to produce and test different glycoforms of the bnAbs PG9, PG16, 10–1074, NIH45–46^G54W^, 10E8, PGT121, PGT128, PGT145, PGT135, in addition to b12 and mutated forms (N92T) of VRC01 (mVRC01) and NIH45–46^G54W^ (mNIH45–46^G54W^), and to assess their in vivo properties in macaques to distinguish those most likely to comprise or become a component of an affordable and efficacious immunotherapeutic cocktail to prevent or control HIV infection in humans.

## Materials and Methods

### Non-human primates

Animals were housed at BIOQUAL's housing facilities in Rockville, MD. Care and husbandry of all non-human primates were provided in compliance with federal laws and guidelines as well as in accordance with recommendations provided in the NIH guide and other accepted standards of laboratory animal care and use. BIOQUAL is accredited by the Association for the Assessment and Accreditation of Laboratory Animal Care, (AAALAC file #624) and holds an Assurance on file with the National Institute of Health, Office for Protection of Research Risks as required by the US Public Health Service Policy on Humane Care and Use of Laboratory Animals. The PHS Animal Welfare Assurance File Number #A-3086–01. Animals were sedated with ketamine or telazol for all technical procedures. Ketamine was given i.m. in amounts necessary for short-term procedures such as blood drawing.

Rhesus macaques (*Macaca mulatta*) were housed at BIOQUAL, Inc. MD, in accordance with the recommendations of the Association for Assessment and Accreditation of Laboratory Animal Care International Standards and with the recommendations in the Guide for the Care and Use of Laboratory Animals of the United States—National Institutes of Health. The Institutional Animal Use and Care Committee of BIOQUAL approved these experiments. When immobilization was necessary, the animals were sedated intramuscularly with 10 mg/kg of Ketamine HCl (Parke-Davis, Morris Plains N.J.) before any direct handling or procedures. All efforts were made to minimize suffering. Details of animal welfare and steps taken to ameliorate suffering were in accordance with the recommendations of the Weatherall report, “The use of non-human primates in research”. Animals were housed in an air-conditioned facility with an ambient temperature of 21–25°C, a relative humidity of 40%–60% and a 12 h light/dark cycle. Animals were socially housed when possible or individually housed if no compatible pairing could be found. The animals were housed in suspended stainless steel wire-bottomed 6 sq ft cages and provided with a commercial primate diet and fresh fruit and vegetables twice daily with water freely available at all times. Social housing, toys, foraging equipment and mirrors were provided. Animals were monitored at least twice daily for behavior, food intake, activity, and overall health by trained technicians. No macaques were euthanized and all animals were returned to the colony for recycling.

### Antibody production in plants

Antibody production by *Agrobacterium*-mediated transient gene expression in *N*. *benthamiana* was performed as described previously [24]. Synthetic codon optimized variable domains were flanked by type-IIs restriction sites and cloned into pTRA plant expression vectors carrying IgG1 and kappa constant domains. The originally published antibody amino acid sequences were used unless indicated otherwise. Antibodies were produced by co-infiltrating 6–8 week old plants or leaves with recombinant *Agrobacteria* suspensions individually carrying the pTRA based heavy and light chain expression plasmids and the pBIN based p19 silencing suppressor from tomato bushy stunt virus. After 10–12 days soluble proteins were extracted and purified by protein-A chromatography producing 100–400 mg/kg, depending on the bnAb. N92T mutated forms of VRC01 and NIH45–46 bnAb were also expressed. For bnAbs mNIH45–46^G54W^ and 10–1074, a C-terminal SEKDEL tag was used for ER retention to generate high mannose glycoforms.

### Macaque studies

Pharmacokinetic and immunogenicity studies of the plant produced HIV mAbs were performed at Bioqual using 3–6 kg Indian rhesus macaques (*Macaca mulatta*) of both sexes, depending on availability. To assess plasma retention of each bnAb following administration, two macaques/ group were injected once or multiple times i.v. with 5, 7.5 or 10 mg/kg doses of each of the mAbs and bled (0.5 ml) from the femoral artery at time zero and for 2–3 weeks at the times indicated time (see figures). Studies using each bnAb were repeated several times. Plasma or serum samples were then tested for both levels of circulating mAb measured by neutralizing antibody activity or by ELISA and also for the induction of anti-human bnAb antibody. Macaque numbers are included in the figures. No macaques became sick during any of the studies.

### Neutralization assays

Neutralizing antibody assays were performed in TZM-bl cells as previously described [25] with purified bnAbs and also with plasma samples collected from macaques at different times following i.v. injection of the bnAbs. Purified recombinant antibodies were tested starting at 50 μg/ml with serial 3-fold dilutions. Plasma (both heat-inactivated and non-heat-inactivated) was tested starting at a 1:20 dilution. Diluted test samples were pre-incubated with pseudovirus (~150,000 relative light unit equivalents) for 1 hr at 37°C before addition of cells. Following 48 hr incubation, cells were lysed and luciferase (Luc) reporter gene activity determined using a microtiter plate luminometer and BriteLite Plus Reagent (Perkin Elmer). Neutralization titers are the sample dilution (for plasma) or antibody concentration (for purified mAb) at which relative luminescence units (RLU) were reduced by 50% compared to RLU in virus control wells after subtraction of background RLU in cell control wells.

For inhibition of neutralization assays in TZM-bl cells, a concentration of mAb that inhibited the target virus at 50–80% was pre-incubated with or without serial dilutions of monkey plasma samples for 1 hr at 37°C prior to adding virus. After an additional 1 hour incubation of mAb/serum/virus, cells were added and the assay was continued according to the standard protocol. The ‘No Serum’ control indicates the level of mAb inhibition of virus. Deviations from this line indicates interference from the plasma sample with neutralization of the mAb.

### ELISA

Two types of ELISAs were used to determine the pharmacokinetics and immunogenicity of the administered bnAbs. To monitor rates of clearance of the circulating bnAbs, 96-well Immuno Module plates (Nunc) were coated with purified plant-derived high mannose 89.6P gp140-KDEL (1 μ g/ml) and incubated for 2 hr at RT with serial dilutions of leaf extracts or purified plant- or mammalian cell-derived mAbs [24]. In some cases e.g. detection of PGT121 levels, wells were coated with anti-human kappa LC (50 mL of 1 μ g/mL) (SIGMA K3502) or with either CHO-derived monomeric HIV BaL-gp120 (NIH HIV Reagent Program) or m.CONgp140 env (a kind gift of Dr Bart Haynes, Duke Univ., NC) which contain mammalian complex glycans required for binding [12]. Control HEK-293 VRC01 was kindly provided by the VRC, NIH and the CHO-derived PGT121 by IAVI, NY. Wells were blocked with 5% (w/v) milk in PBST, washed 3–5 times with PBST, incubated with a 1/8,000 dilution of peroxidase-labeled goat anti-human IgG (Fc) (A0170, Sigma), and developed with tetramethylbenzidine (TMB) liquid substrate system (T0440, Sigma Chemical Co, MO). Reactions were stopped with 0.5 N H_2_SO_4_, and endpoints were determined at 450 nm using the SPECTRA max PLUS plate reader (Molecular Devices). Due to the variability in the background of individual macaques, the initial prebleed OD450 values were subtracted.

To monitor the presence of a macaque antibody response against the injected human HIV bnAbs, ELISA plates were coated at RT with the target antibodies at 1.2μ g/ml for 2–4 hr. Following incubation, wells were blocked, washed and incubated for 2 hr with monkey plasma or serum samples at 1/500 and 1/2500 dilutions followed by a third 2 hr incubation with 1/4,000 of mouse anti-macaque IgG (1B3-HRP, Nonhuman Primate Reagent Resource).

### Antibody autoreactivity against human host cellular antigens

Reactivity testing of plant-derived bnAb, starting at 50μ g/ml, was kindly performed by Krissy Lloyd (Duke University) against a panel of 9 autoantigens associated with presence of autoimmune diseases (Sjogren’s syndrome antigens A and B, Smith antigen, ribonucleoprotein, centromere B, histone, scleroderma 70, Jo-1 proteins and dsDNA). CH98 reacted with dsDNA using 4E10 and 2F5 as controls while all plant bnAbs were negative (data not shown).

## Results

### Neutralization activity of plant-derived bnAbs

**Fig 1:**
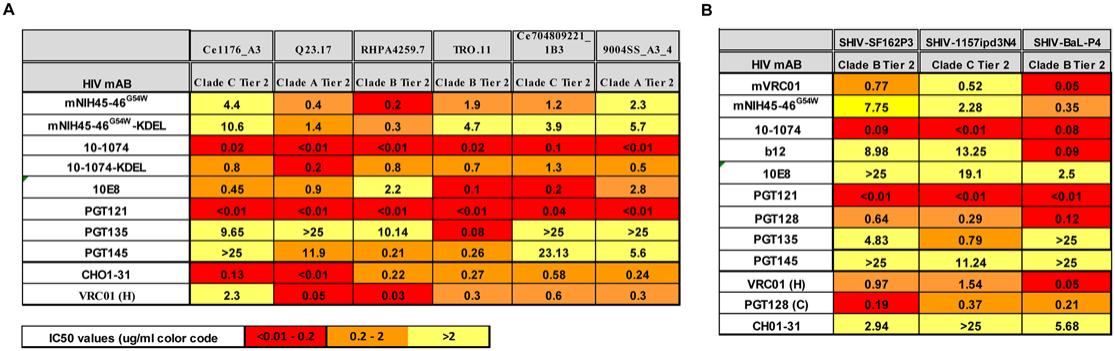
Neutralization titers of *N*.*benthamiana*-derived bnAbs and control bnAbs against six Tier 2 HIV isolates (A) and against three SHIV isolates (B) using pseudovirus-based TZM-bl cells. IC50 values are color coded as indicated. Control bnAb produced in CHO (C) and VRC01 produced in HEK293 (H) cells. CHO1–31 is a pool of two CHO-1 (PG9-like) and CHO-31 (VRC01-like) bnAbs which strongly neutralizes all HIV isolates.

Neutralization activity of KDEL-tagged (usually high mannose) and non-KDEL- tagged (usually complex) glycoforms of *N*.*b/p19*-derived mAb (Fig. 1) was measured in a TZM-bl assay as a reduction in Luc reporter gene expression after a single round of infection with a small panel of Env-pseudotyped viruses, including six Tier 2 HIV-1 isolates (Fig. 1a) and three simian/human HIV (SHIV) isolates (Fig. 1b). Several bnAbs produced in mammalian cells (VRC01(H) produced in HEK293 cells, PGT128 (C) produced in CHO cells, and CHO1–31, a pool of two mAbs, known to efficiently neutralize most HIV variants) were used as positive controls. Overall, with the exception of NIH45–46^**G54W**^, all plant-derived bnAb IC50s were similar to control mAbs; PGT121 exhibiting the highest potency with an IC50 of <0.01 against 8 out of 9 HIV and SHIV isolates. Surprisingly, the high mannose glycoforms, 10–1074-KDEL and NIH45–46^G54W^-KDEL had lower neutralization activity than the non-KDEL complex glycoforms. Potency against SHIV isolates SF162P3, 1157ipd3N4 and SHIV-Bal-P4 was assessed to determine the most appropriate challenge isolate for macaque passive protection studies. 10–1074 and PGT121 had the lowest IC50 against the clade C 1157ipd3N4. Despite high expression levels, the IC50s of PG9 and PG16 were very high due to undersulfation of tyrosines within their CDR-H3 regions resulting from no/low tyrosylprotein sulfotransferase gene expression in plants (not shown). Only those antibodies that showed low IC50 and good expression levels were taken further into in vivo studies.

### Pharmacokinetics of plant-derived bnAbs

The efficacy of any immunotherapeutic clearly depends on circulatory stability. Since the breadth and potency of HIV bnAbs correlate closely with their level of somatic mutation and the frequency of indels (insertions /deletions) [26–30], it was critical to determine how H and L chain mutations, long heavy chain complementarity-determining region 3 (HCDR3) and, in this particular study, plant-specific glycosylation, would impact their plasma retention time, as well as their immunogenicity, following in vivo administration.

**Fig 2:**
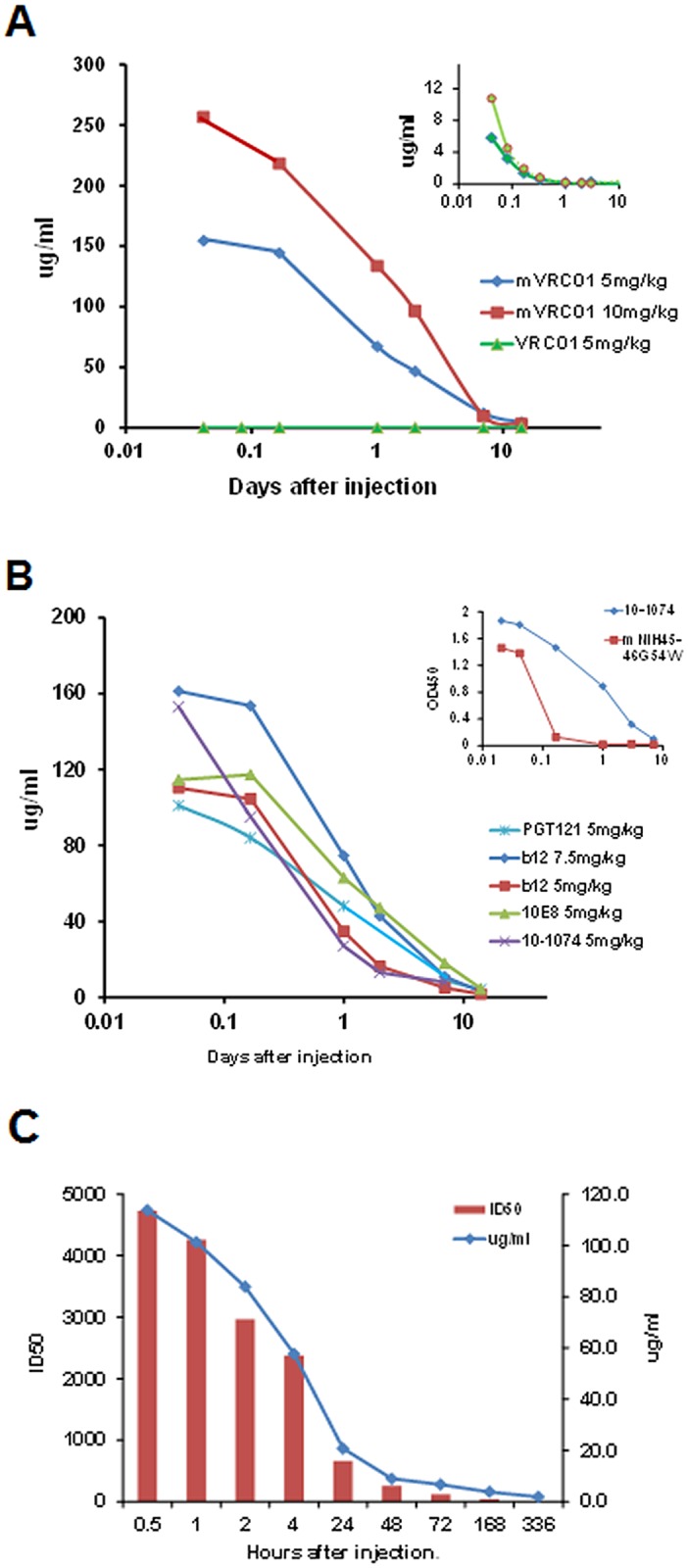
Circulatory clearance of bnAbs in macaques at different times after i.v. injection as measured by ELISA. Data are an average of two macaques. Each pharmacokinetics study was repeated once or twice. (A) Plasma levels (μ g/ml) following injection of mVRC01 at 5mg/kg (blue) or 10mg/kg (red) and VRC01 (10mg/kg, green). The insert shows clearance of the non-mutated VRC01 in each of two macaques on a different scale. (B) Average μ g/kg of two macaques injected with b12 (5 and 7.5 mg/kg), 10E8, 10–1074, PGT121 (each at 5mg/kg). The insert shows a comparison between 10–1074 and mNIH45–46^G54W^ both injected at 5mg/kg (C) Comparison of clearance of PGT121 (5mg/kg) as measured by ELISA (μ g/ml) and a pseudovirus-based TZM-bl assay against the RHPA4259.7 isolate (ID50) at different days following i.v. injection into macaques.

To assess in vivo stability, each plant-derived bnAb was injected i.v. into two macaques at 5, 7.5 or 10 mg/kg, and circulating bnAb levels monitored by ELISA using wells coated with either plant-derived (high mannose) HIV 89.6P gp140deltaCFI envelope, or in the case of PGT121, with either CHO-derived Bal.gp120 or M.CON-Sgp140 envelope, providing the specific PGT121 complex glycan epitopes terminating in galactose and sialic acid. Despite known animal variability, most bnAbs exhibited Cmax levels of ~110–150 μ g/ml in the circulation ~1hr following a 5mg/kg i.v. injection; consistent with plasma representing ~4% of the body weight. One exception shown in Fig. 2a was wild type VRC01 which, as a result of an L chain glycan at N92, was rapidly cleared by 4 hours with an average Cmax of ~8μ g/ml (insert, Fig. 2a). To overcome this rapid clearance, the mutated form N92T of both VRC01 (mVRC01) and its family member NIH45–46^G54W^ (mNIH45–46^G54W^) were produced and tested. Following injection, mVRC01 now exhibited a more typical dose-dependent Cmax of 260 μ g/ml and 160 μ g/ml in the circulation 1hr following injections of 10mg/kg and 5mg/kg i.v. respectively.

Fig. 2b indicates the pharmacokinetics of clearance of b12, 10-1074, 10E8 and m45–46^**G54W**^. Unlike VRC01, plasma levels of m45–46^**G54W**^ were reduced rapidly despite the N92T mutation (insert Fig. 2b). Surprisingly, the pharmacokinetics of PGT121 in the present study yielded varying results. Thus, while some monkeys injected with 5mg/kg exhibited pharmacokinetics similar to those in Fig. 2a (not shown), other monkeys exhibited rapid clearance when monitored using either ELISA (μ g/ml) or neutralizing (ID50) assays (Fig. 2c).

### Immunogenicity of human HIV bnAbs

**Fig 3:**
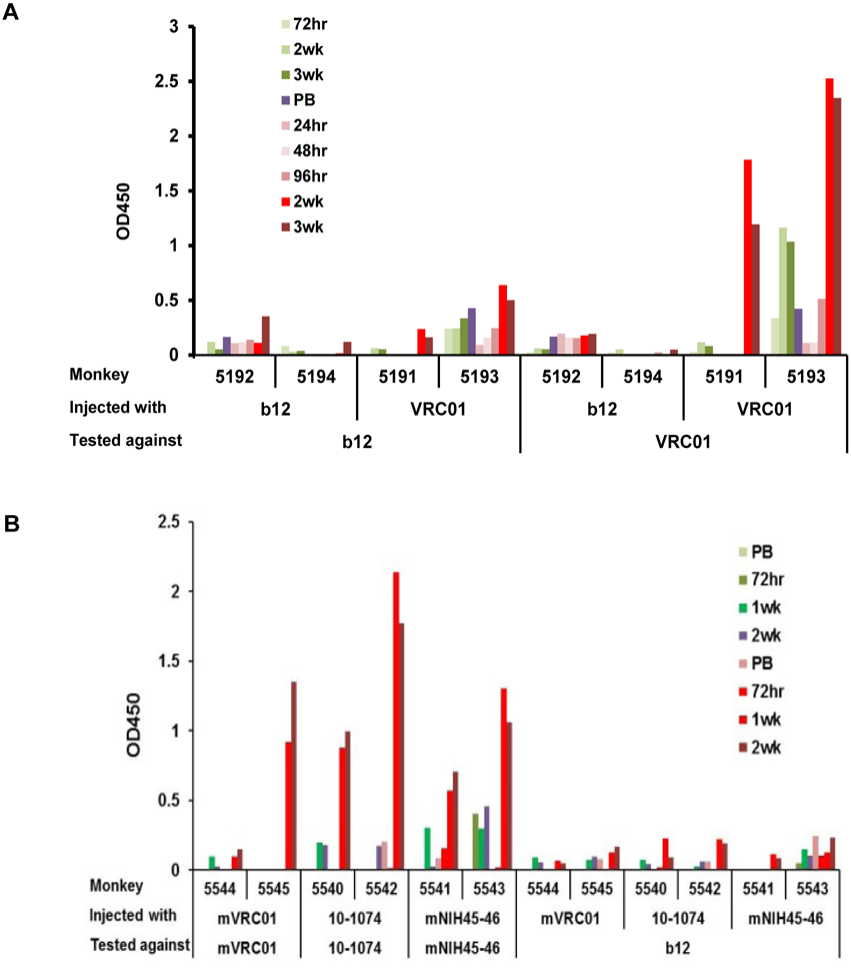
Immunogenicity of bnAbs in macaques following two injections two weeks apart. Plasma samples collected before injection (PB) and at the time indicated from macaques receiving (A) VRC01 (#5191 and #5193) or b12 (#5194 and #5192) and (B) mVRC01 (#5544 and #5545), 10-1074 (#5540 and #5542) and mNIH45–46^G54W^ (#5541 and #5543). Each sample was assayed by ELISA against the injected bnAb and, as a control against, b12 to demonstrate anti-bnAb antibodies.

Since passive immunotherapy may involve multiple administrations of highly mutated mAbs, immunogenicity was also assessed in parallel with the pharmacokinetic studies following two or three injections of plant-derived VRC01, mVRC01, 10–1074, NIH45–46^G54W^, b12 and PGT121 bnAbs (5–10 mg/kg) administered 2–3 weeks apart. To measure the monkey anti-human antibody responses against the injected bnAbs, plasma was tested at different times after each injection using an ELISA that employed an anti-monkey secondary antibody (1B3) that does not cross react with human IgG. Three types of responses occurred: 1. no response, which indicated that the particular bnAb is not highly immunogenic. 2. primary responses at 3–14 days after the first injection reflecting possible polyreactivity or environmental stimulation and 3) clear secondary anti-human IgG responses induced 7–21 days after the second injection. In the first immunogenicity study, in which two macaques each received either 4.5 and 7.5 mg/kg of b12 (#5192, #5194) or 4.5 or 10mg/kg of VRC01 (#5191, 5193), all three types of responses were evident. Fig. 3A shows the binding of plasma from each of the four animals to both b12- and VRC01-coated wells and demonstrates that while b12 exhibited no/low anti-b12 responses, both macaques receiving VRC01 made a substantial anti-VRC01 antibody response at 14–21 days following the second administration. These anti-VRC01 antibodies did not cross react with b12 although plasma from macaque #5193, which appeared to be previously stimulated, did exhibit some cross-reactivity with b12.

In the second immunogenicity study, the potent highly mutated bnAbs 10–1074 and mNIH45–46^G54W^ were also tested with VRC01 for immunogenicity in macaques following two i.v. injections of 5mg/kg 2–3 weeks apart. Fig. 3B shows that except for macaque #5544, which was injected with mVRC01, each monkey exhibited moderate to high anti-human bnAb responses 7–14 days after the second injection. Again, none of the plasma from these 6 macaques reacted with b12. Interestingly, the potent highly mutated PGT121, elicited no anti-antibody response against PGT121 following three injections of 5 mg/kg into two macaques (study #1) and two injections into two macaques (study #2) despite belonging to the same family as 10–1074 (data not shown). In these studies, monkey plasma that cross-reacts with 10–1074 was used as a positive control.

**Fig 4:**
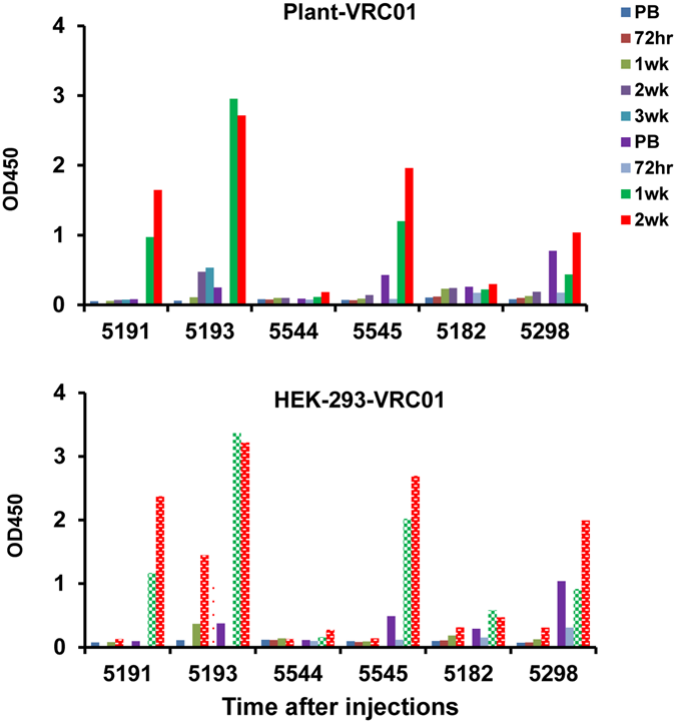
Comparison of plasma levels of anti-VRC01 antibody in all macaques injected twice with VRC01 by ELISA using plant-derived VRC01 (A) and HEK293-derived VRC01 (B). The numbers of the six macaques injected (2 macaques/gp in three different studies) are indicated and the times of the bleeds are color coded.

To demonstrate the antibody response was not specific for contaminants in the plant bnAb preparations (e.g. host cell derived impurities), plasma from all 6 of the VRC01-injected macaques were also tested by ELISA using highly purified HEK293-derived VRC01. Plasma collected at different times from VRC01-injected macaques exhibited identical binding to wells coated with either highly purified HEK293- or plant-derived VRC01 demonstrating that the observed responses were indeed specifically directed at the bnAbs (Fig. 4).

**Fig 5:**
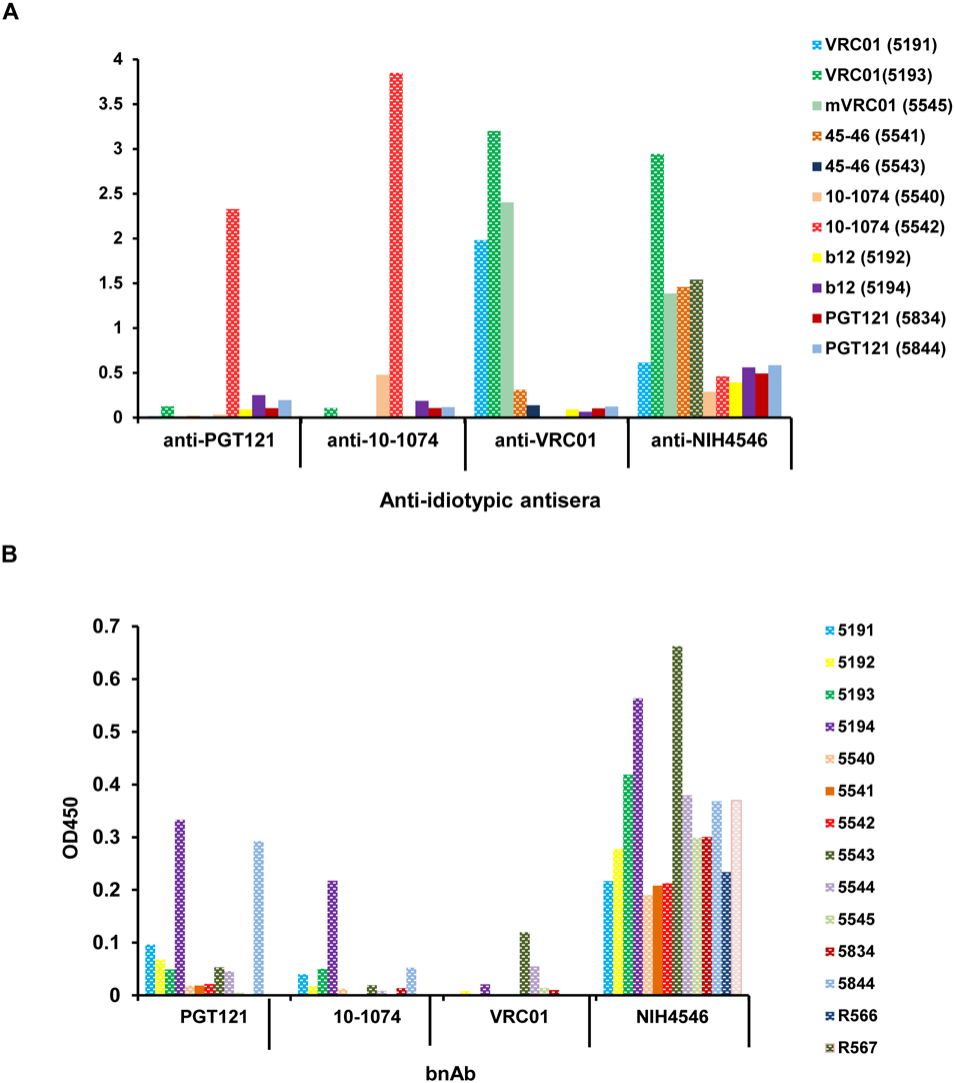
Reactivity of plasma from macaques injected with different bnAbs. (A) Plasma samples from the 11 macaques injected (color coded) were collected 2–3 weeks following the second injection and idiotype specificity was demonstrated by ELISA against PGT121, 10–1074, VRC01 and NIH45–46 produced in mammalian cells. (B) Screening of 14 naïve macaques for pre-existing reactivity against PGT121, 10–1074, VRC01 and NIH45–46^G54W^ prior to injection.

To confirm the anti-idiotypic specificity of the antibodies induced by VRC01, mVRC01, 10–1074 and mNIH45–46^G54W^, the positive sera from the macaques collected at 14–21 days after the second injection of each bnAb were tested using an ELISA against HEK-293-derived VRC01 and CHO-derived 10–1074, NIH45–46^G54W^ and PGT121. The results in Fig. 5A indicate the plasma from each macaque binds strongly only to the specific bnAb it received; any cross reactivity observed being explainable by the clonal families to which they belong. Thus, sera from macaques administered VRC01 or mVRC01 bound only to VRC01 and the clonally related NIH45–56^G54W^. However antibodies induced by two injections of NIH45–46^G54W^ bound strongly to VRC01, moderately to itself and weakly, albeit significantly, to all bnAbs tested; suggesting that these anti-idiotypic antibodies were directed to either the TARDY insertion in the CDRH3 region and/or the 6-amino acid changes in the CDR1 and CDR2. Importantly, while macaques injected with PGT121 did not exhibit anti-PGT121 antibodies, plasma from animals injected twice with 10–1074 contained antibodies specific for both 10–1074 as well as the related family member PGT121. No monkey plasma showed significant binding to b12 or 10E8 (not shown) and all anti-idiotypic antibodies tested showed similar binding patterns to either mammalian- and plant-derived bnAbs. It is important to note that many macaques appeared to have pre-existing “anti-idiotypic” antibodies in their circulation prior to injection; presumably due to polyreactivity or environmental stimulation. Fourteen naïve macaques were tested for pre-existing reactivities to PGT121, VRC01, 10–1074 and NIH45–46 by ELISA. The results in Fig. 5B illustrate two points: (i) similar to the cross-reactivity previously observed (macaque #5193 in Fig. 3A), most monkeys tested had low to moderate levels of antibodies specific for NIH45–46^G54W^ and (ii) sera from some macaques e.g. #5191, 5194 and 5844 bound at varying levels to several of the bnAbs tested.

**Fig 6:**
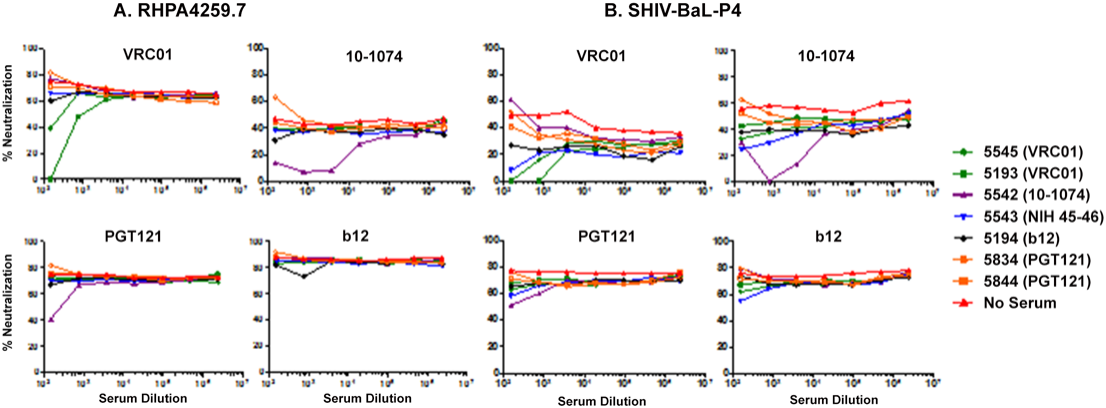
Inhibition of VRC01, 10–1074, b12 and PGT121 neutralizing activity in the TZM-bl assay against HIV RHPA4259.7 and SHIV-BaL-P4 by different macaque anti-idiotypic antibodies. Concentrations of each of the bnAb that inhibited the target virus at 50–80% was pre-incubated with or without serial dilutions of 7 different monkey plasma samples (color coded) prior to adding virus. Deviations from this “no serum” control line indicates interference from the macaque sample with neutralization of the bnAb.

Finally, to corroborate the binding assays and to evaluate whether the macaque anti-idiotypic antibodies could functionally inhibit their cognate idiotype, dilutions of each of 7 plasma from monkeys injected twice with VRC01, 10–1074, b12 and PGT121 were assessed for their ability to inhibit neutralization by each of the four bnAbs when tested against HIV RHPA4259.7 and SHIV-Bal-P4 isolates in a pseudovirus/TZM-bl assay. Fig. 6 demonstrates that the inhibition by each of the anti-idiotypic antibodies was highly specific, dose dependent and effective against both isolates. Thus, as observed in the binding assays, (i) anti-VRC01 blocked neutralization of both HIV and SHIV by VRC01, although it did not significantly cross-block NIH45–46 neutralization of the SHIV-Bal-P4 (ii) anti-10–1074 inhibited strongly 10–1074 and to a lesser extent PGT121 and (iii) in agreement with Fig. 5a, plasma from PGT121- and b12- injected macaques did not inhibit their cognate idiotypes.

## Discussion

Recently discovered bnAbs have been primarily selected based on their extraordinary breadth and potency of neutralization. However, as observed in the current study, plasma stability and immunogenicity may determine their potential as therapeutics. Thus, rigorous in vivo screening and evaluation of these highly promising bnAbs is a mandatory exercise towards their development as potential therapeutics. Here we have used the transient plant *Nb*/p19 system to produce several bnAbs and tested their in vivo properties in macaques.

The impact of an L-chain glycan at N92 on the clearance from the blood was seen during PK studies with plant-derived VRC01. In this case, injection of either 4.5 mg/kg or 10 mg/kg of the original VRC01 exhibited very low Cmax at 30 mins and total clearance by 4 hr (insert in Fig. 3A) compared with a typical dose dependent Cmax and greatly increased retention time when the L-chain glycan was eliminated (mVRC01). Unlike the unexposed glycan at N297 in the CH2 chain domain of IgG antibodies, which to date has been shown to exert either no effect on clearance rates or a reduction in circulatory retention in the case of Man5 appended glycans, these results suggest that exposed glycans terminating in GlcNAc, which make up a majority of plant-derived proteins, lead to rapid receptor-mediated removal. Interestingly, in contrast to the mVRC01, mNIH45–46^**G54W**^ (N92T) was cleared rapidly despite the elimination of the L chain glycan. Overall, while there was some variability between of individual plant-derived bnAb, the observed profiles were similar to those seen for CHO-derived molecules [31].

Unlike antibodies elicited through vaccination, the breadth and potency of broadly neutralizing mAbs have been shown to correlate with their level of somatic mutation and frequency of insertions, especially in their distinctively long CDR3 regions. In this context, VRC01 VH and VL differ from their germ lines by 32% and 17% respectively, 10–1074 VH by 29%, NIH45–46 VH and VL by 40% and 26%) and PGT121 VH and VL by 34% and 28%, while b12 is the least mutated (~14%) [26–30]. The 2–3 year-maturation process whereby these antibodies develop, or fail to develop, from germ line to affinity matured antibody, are only now being elucidated. While the majority of HIV-infected individuals do not generate the mutations in their anti-HIV env B cell repertoire required for neutralization breadth and potency, others may produce highly mutated antibodies e.g. the anti-gp41 2F5 and 4E10, but due to their polyreactivity and /or autoreactivity, may be deleted or rendered tolerant [32–34].

In the present studies, the absence of binding to the 9 autoantigens tested (data not shown) and the anti-idiotypic antibody response observed in macaques following the second injection of the highly mutated bnAbs raises the another intriguing possibility that the HIV-infected individuals who lack broadly neutralizing antibody in their circulation, may have actually generated potent anti-HIV responses at some stage, but may have subsequently induced an anti-idiotypic response leading to their elimination and overall reduced plasma neutralizing activity.

While a close association between the levels of mutation and immunogenicity was initially apparent with b12, mVRC01, 10–1074 and NIH45–46^G54W^, the highly mutated PGT121 was not found to be immunogenic after two or three injections two weeks apart. The absence of immunogenicity of b12 is consistent with previous macaque studies where multiple administrations of b12 did not appear to induce an anti-b12 response [6]. This lack of PGT121 immunogenicity was interesting, since10–1074, which is clonally related, induced a strong antibody response in 10–1074-injected macaques which cross-reacted with PGT121 by ELISA and to a lesser extent by neutralization inhibition. Based on the large number of common substitutions (50%) in the VL chains of these two molecules [12], it is possible that these residues represent the antigenic epitope/s on 10–10–74 and PGT121 responsible for the cross-reactivity in the ELISA assays, while the lack of immunogenicity of PGT121 may be a result of either specific mutations in the more diverse CDRVH, CDRL3 and framework regions [29] or the absence of a T cell epitope in the PGT121. Identification of novel bnAbs with reduced immunogenicity will be expedited by the longitudinal studies currently being employed to search for family members of known bnAbs e.g. VRC01 and PGT121, that retain neutralization breadth and potency but with limited somatic mutation [35,36].

In vivo testing with plant-derived NIH45–46^**G545W**^ revealed that this bnAb has many features different than the other bnAbs tested, which may reflect polyreactivity [32]. For example, most macaques appear to have varying levels of pre-existing antibodies that bind to mNIH45–46^G54W^ prior to any exogenous administration when tested on both plant-and CHO-derived molecules. The presence of these antibodies is also consistent with the comparatively more rapid clearance of NIH45–46^G54W^ from the circulation following i.v. injection in both macaques (Fig. 2B insert) and humanized mice [37].

The observed immunogenicity is not a result of plant production of the bnAbs for several reasons; (i) optimization for plant expression, did not involve any amino acid changes; (ii) although the monkey anti-human antibody responses observed could be directed to IgG Fc regions, it is unlikely since all the bnAbs produced share the same constant regions while only 3/5 induced anti-idiotypic antibodies. (iii) antibodies to plant-specific glycans, i.e. β(1,2)-xylose, α(1,3)-fucose, also did not seem to play a role since all bnAbs, both immunogenic and non-immunogenic, are produced in the same *N*.*benthamiana* host plants using the same glycosylation machinery; (iv) strong anti-VRC01 responses were observed in 5/6 macaques (#5544 being non-responsive to VRC01 indicating a genetic component), when in vivo clearance was rapid (WT) or normal (N92T mutation); and (v) similar binding patterns of the anti-VRC01 idiotypic antisera to both highly purified mammalian (HEK293-derived) and to plant bnAbs were obtained. It is also unlikely that the immunogenicity simply results from the injection of human proteins into macaques since two of the bnAbs, PGT121 and b12, were not immunogenic in macaques.

The finding that a relatively high number of the bnAbs tested exhibited immunogenicity following multiple injections in macaques suggests that engineering of the current bnAbs or the continued development of new bnAbs for sustaining potent antibody-based therapies may be required for use in humans. The rapid production of 15 broadly neutralizing plant-derived HIV mAbs in the current study highlights the unique advantages of the transient plant system in terms of speed and versatility, pathogen-free nature and low-tech requirements; particularly in the early developmental stages from “cloning to preclinical protection studies”.

Taken together, the current studies demonstrate that anti-idiotypic antibodies induced in monkeys are capable of strongly binding to and inhibiting neutralization of their cognate idiotypes and to a lesser extent their close family members. The findings in macaques for 10–1047 and PGT121 suggest that specific mutations, rather than the level of mutations per se, in these bnAbs contribute to their immunogenicity and call attention to the prospect that mutated bnAbs will be immunogenic in humans, thereby reducing their value for prophylaxis and therapy of HIV-1 involving multiple admin-istrations. The results also stress the need to perform these macaque studies on a case-by-case basis and also to monitor the production of anti-idiotypic antibody responses in recipients of passive HIV bnAb therapeutics in human clinical trials.

## References

[1] Walker LM, Phogat SK, Chan-Hui PY, Wagner D, Phung P, Goss JL, et al Broad and potent neutralizing antibodies from an African donor reveal a new HIV-1 vaccine target. Science. 2009; 326(5950):285–289. 10.1126/science.1178746 19729618 PMC3335270

[2] Scheid JF, Mouquet H, Feldhahn N, Seaman MS, Velinzon K, Pietzsch J. et al Broad diversity of neutralizing antibodies isolated from memory B cells in HIV-infected individuals. Nature. 2009; 458(7238):636–640. 10.1038/nature07930 19287373

[3] Kwong PD, Mascola JR, Nabel GJ. Mining the B cell repertoire for broadly neutralizing monoclonal antibodies to HIV-1. Cell Host Microbe. 2009; 6:292–294. 10.1016/j.chom.2009.09.008 19837366 PMC8513376

[4] Mascola JR, Lewis MG, Stiegler G, Harris D, VanCott TC, Hayes D, et al Protection of Macaques against pathogenic simian/human immuno-deficiency virus 89.6PD by passive transfer of neutralizing antibodies. J Virol. 1999; 73(5):4009–4018. 10196297 10.1128/jvi.73.5.4009-4018.1999 PMC104180

[5] Parren PW, Marx PA, Hessell AJ, Luckay A, Harouse J, Cheng-Mayer C, et al Antibody protects macaques against vaginal challenge with a pathogenic R5 simian/human immunodeficiency virus at serum levels giving complete neutralization in vitro. J Virol. 2001; 75(17):8340–8347. 11483779 10.1128/JVI.75.17.8340-8347.2001 PMC115078

[6] Hessell AJ, Poignard P, Hunter M, Hangartner L, Tehrani DM, Bleeker WK, et al Effective, low-titer antibody protection against low-dose repeated mucosal SHIV challenge in macaques. Nat Med. 2009; 15(8):951–954. 10.1038/nm.1974 19525965 PMC4334439

[7] Shingai M, Nishimura Y, Klein F, Mouquet H, Donau OK, Plishka R, et al Antibody-mediated immunotherapy of macaques chronically infected with SHIV suppresses viraemia. Nature. 2013; 503(7475):277–280. 10.1038/nature12746 24172896 PMC4133787

[8] Gorny MK, Zolla-Pazner S. Immunoprophylaxis against mother-to-child transmission of HIV-1. PLoS Med. 2006; 3(7): e259 16842019 10.1371/journal.pmed.0030259 PMC1513047

[9] Trkola A, Kuster H, Rusert P, Joos B, Fischer M, Leemann C, et al Delay of HIV-1 rebound after cessation of antiretroviral therapy through passive transfer of human neutralizing antibodies. Nat Med. 2005; 11(6): 615–622. 15880120 10.1038/nm1244

[10] Klein F, Mouquet H, Dosenovic P, Scheid JF, Scharf L, Nussenzweig. Antibodies in HIV-1 vaccine development and therapy. Science. 2013; 341(6151): 1199–1204. 10.1126/science.1241144 24031012 PMC3970325

[11] Barouch DH, Whitney JB, Moldt B, Klein F, Oliveira TY, Liu J, et al Therapeutic efficacy of potent neutralizing HIV-1-specific monoclonal antibodies in SHIV-infected rhesus monkeys. Nature. 2013; 1503(7475):224–228. 10.1038/nature12744 24172905 PMC4017780

[12] Mouquet H, Scharf L, Euler Z, Liu Y, Eden C, Scheid JF, et al Complex-type N-glycan recognition by potent broadly neutralizing HIV antibodies. Proc Natl Acad Sci USA. 2012; 109(47):3268–3277. 10.1073/pnas.1217207109 23115339 PMC3511153

[13] Zhou T, Georgiev I, Wu X, Yang ZY, Dai K, Finzi A, et al Structural basis for broad and potent neutralization of HIV-1 by antibody VRC01. Science. 2010; 329(5993): 811–817. 10.1126/science.1192819 20616231 PMC2981354

[14] Pancera M, McLellan JS, Wu X, Zhu J, Changela A, Schmidt SD, et al Crystal structure of PG16 and chimeric dissection with somatically related PG9:structure-function analysis of two quaternary-specific antibodies that effectively neutralize HIV-1. J Virol. 2010; 84(16): 8098–8110. 10.1128/JVI.00966-10 20538861 PMC2916520

[15] Walker LM, Huber M, Doores KJ, Falkowska E, Pejchal R, Julien JP, et al Broad neutralization coverage of HIV by multiple highly potent antibodies. Nature. 2011; 477(7365): 466–470. 10.1038/nature10373 21849977 PMC3393110

[16] Diskin R, Scheid JF, Marcovecchio PM, West AP Jr, Klein F, Gao H, et al Increasing the potency and breadth of an HIV antibody by using structure-based rational design. Science. 2011; 334(6060):1289–1293. 10.1126/science.1213782 22033520 PMC3232316

[17] Scharf L, Scheid JF, Lee JH, West AP Jr, Chen C, Gao H, et al Antibody 8ANC195 reveals a site of broad vulnerability on the HIV-1 envelope spike. Cell Rep. 2014; 7(3):785–795. 10.1016/j.celrep.2014.04.001 24767986 PMC4109818

[18] Huang J, Ofek G, Laub L, Louder MK, Doria-Rose NA, Longo NS, et al Broad and potent neutralization of HIV-1 by a gp41-specific human antibody. Nature. 2012; 491(7424):406–412. 10.1038/nature11544 23151583 PMC4854285

[19] Sok D, Laserson U, Laserson J, Liu Y, Vigneault F, Julien JP, et al The Effects of Somatic Hypermutation on Neutralization and Binding in the PGT121 Family of Broadly Neutralizing HIV Antibodies. PLoS Pathog. 2013; 9(11): 1–20. 10.1371/journal.ppat.1003754 PMC3836729 24278016

[20] Matucci A, Petroni G, Pratesi S, Nencini F, Maggi E, Vultaggio A. Immunogenicity of biological agents: Basic Knowledge and Clinical Implications. International trends in immunity. 2014; 2(1):11–16.

[21] Gomord V, Chamberlain P, Jefferis R, Faye L Biopharmaceutical production in plants: problems, solutions and opportunities. Trends Biotechnol. 2005; 23(11): 559–565. 16168504 10.1016/j.tibtech.2005.09.003

[22] Stoger E, Fischer R, Moloney M, Ma JK. Plant molecular pharming for the treatment of chronic and infectious diseases. Annu Rev Plant Biol. 2014; 65:743–768. 10.1146/annurev-arplant-050213-035850 24579993

[23] Ramessar K, Rademacher T, Sack M, Stadlmann J, Platis D, Stiegler G, et al Cost effective production of a vaginal protein microbicide to prevent HIV transmission. Proc Natl Acad Sci USA. 2008; 105(10): 3727–3732. 10.1073/pnas.0708841104 18316741 PMC2268773

[24] Rosenberg Y, Sack M, Montefiori D, Forthal D, Mao L, Hernandez-Abanto S, et al Rapid high-level production of functional HIV broadly neutralizing monoclonal antibodies in transient plant expression systems. PLoS One. 2013; 8(3):e58724 10.1371/journal.pone.0058724 23533588 PMC3606348

[25] Montefiori DC. Measuring HIV neutralization in a luciferase reporter gene assay. Methods Mol Biol. 2009; 485:395–405. 10.1007/978-1-59745-170-3_26 19020839

[26] Wu X, Yang ZY, Li Y, Hogerkorp CM, Schief WR, Seaman MS, et al Rational design of envelope identifies broadly neutralizing human monoclonal antibodies to HIV-1. Science. 2010; 329(5993):856–861. 10.1126/science.1187659 20616233 PMC2965066

[27] Walker LM, Huber M, Doores KJ, Falkowska E, Pejchal R, Julien JP, et al Broad neutralization coverage of HIV by multiple highly potent antibodies. Nature. 2011; 477(7365):466–470. 10.1038/nature10373 21849977 PMC3393110

[28] Scheid JF, Mouquet H, Ueberheide B, Diskin R, Klein F, Oliveira TY, et al Sequence and structural convergence of broad and potent HIV antibodies that mimic CD4 binding. Science. 2011; 333(6049):1633–1637. 10.1126/science.1207227 21764753 PMC3351836

[29] Klein F, Diskin R, Scheid JF, Gaebler C, Mouquet H, Georgiev IS, et al Somatic mutations of the immunoglobulin framework are generally required for broad and potent HIV-1 neutralization. Cell. 2013; 153(1):126–138. 10.1016/j.cell.2013.03.018 23540694 PMC3792590

[30] Xiao X, Chen W, Feng Y, Zhu Z, Prabakaran P, Wang Y, et al Germline-like predecessors of broadly neutralizing antibodies lack measurable binding to HIV-1 envelope glycoproteins: implications for evasion of immune responses and design of vaccine immunogens. Biophys Res Commun. 2009; 390(3):404–409. 10.1016/j.bbrc.2009.09.029 19748484 PMC2787893

[31] Moldt B, Rakasz EG, Schultz N, Chan-Hui PY, Swiderek K, Weisqrau KL, et al Highly potent HIV-specific antibody neutralization in vitro translates into effective protection against mucosal SHIV challenge in vivo. Proc Natl Acad Sci U S A. 2012;109(46):18921–18925. 10.1073/pnas.1214785109 23100539 PMC3503218

[32] Liao HX, Chen X, Munshaw S, Zhang R, Marshall DJ, Vandergrift N, et al Initial antibodies binding to HIV-1 gp41 in acutely infected subjects are polyreactive and highly mutated. J Exp Med. 2011; 208(11):2237–2249. 10.1084/jem.20110363 21987658 PMC3201211

[33] Yang G, Holl TM, Liu Y, Li Y, Lu X, Nicely NI, et al Identification of autoantigens recognized by the 2F5 and 4E10 broadly neutralizing HIV-1 antibodies. J Exp Med. 2013; 210(2): 241–256. 10.1084/jem.20121977 23359068 PMC3570098

[34] Haynes BF, Fleming J, St Clair EW, Katinger H, Stiegler G, Kunert R, et al Cardiolipin polyspecific autoreactivity in two broadly neutralizing HIV-1 antibodies. Science. 2005; 308(5730):1906–1908. 15860590 10.1126/science.1111781

[35] Moore PL, Gray ES, Wibmer CK, Bhiman JN, Nonyane M, Sheward DJ, et al Evolution of an HIV glycan-dependent broadly neutralizing antibody epitope through immune escape. Nat Med. 2012; 18:1688–1692. 10.1038/nm.2985 23086475 PMC3494733

[36] Doria-Rose NA, Schramm CA, Gorman J, Moore PL, Bhiman JN, DeKosky BJ, et al Developmental pathway for potent V1V2-directed HIV-neutralizing antibodies. Nature. 2014; 509(7498):55–62. 10.1038/nature13036 24590074 PMC4395007

[37] Klein F, Halper-Stromberg A, Horwitz JA, Gruell H, Scheid JF, Bournazos S, et al HIV therapy by a combination of broadly neutralizing antibodies in humanized mice. Nature. 2012; 492(7427):118–122. 10.1038/nature11604 23103874 PMC3809838

